# Supplementary material for: Relevance of Medullary Vein Sign in Neurosarcoidosis
Source: Neurol Int. 2022 Aug 14;14(3):638–47. doi: 10.3390/neurolint14030052 (PMC9397064; doi:10.3390/neurolint14030052)
Supplement: Supplementary file 1 [file neurolint-14-00052-s001.zip › neurolint-1860070-supplementary.pdf]

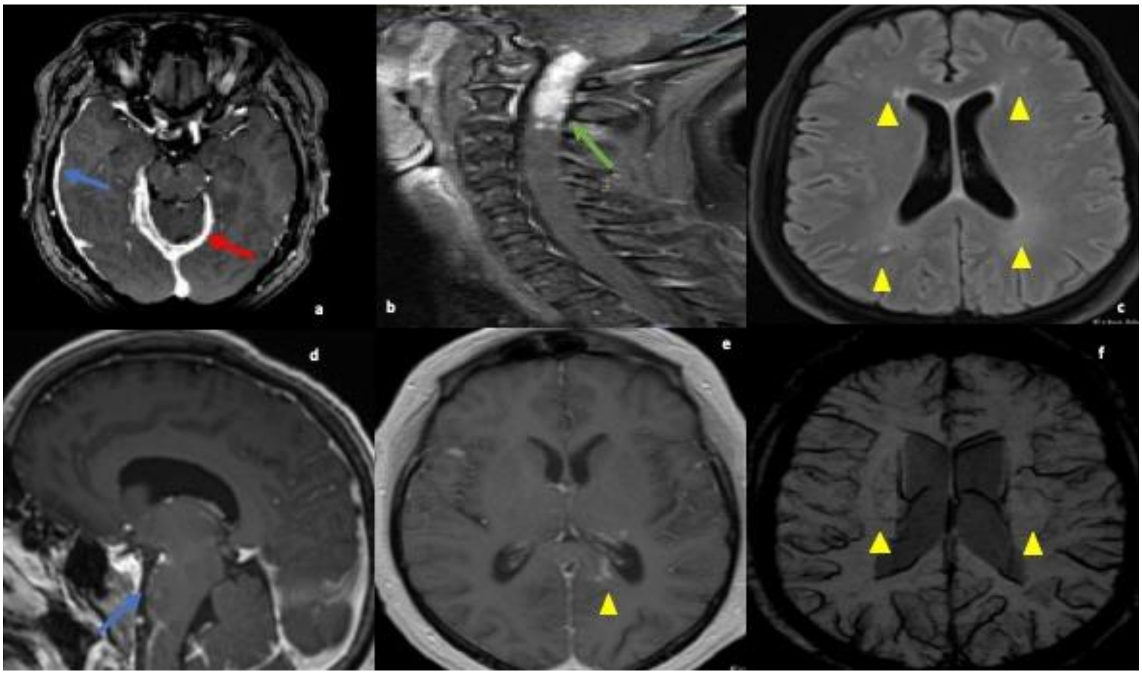

Figure S1. MRI axial T1 images 1(**a**) of the gadolinium-enhanced brain demonstrating dural thickening and enhancement of the right tentorium and cerebellopontine angle (blue and red arrow); 1(**b**) abnormal enhancement in cervicomedullary junction of cord (green arrow); 1(**c**) axial FLAIR-weighted MRI brain reveals hyperintense signal changes in atrium and frontal white matter bilateral (yellow arrowhead). 1(**d**,**e**) MRI brain sagittal and axial T1 post contrast images reveal leptomeningeal enhancement in basilar portion (blue arrow) and right atrium (yellow arrowhead); 1(**f**), and SWI images showing mild dilated medullary vein (yellow arrowhead).
